# Development and psychometric properties of the hospitalized elder abuse questionnaire (HEAQ): a mixed methods study

**DOI:** 10.1186/s12877-022-03400-0

**Published:** 2022-08-30

**Authors:** Zeinab Naderi, Sakineh Gholamzadeh, Abbas Ebadi, Ladan Zarshenas

**Affiliations:** 1 Department of Nursing, Sirjan School of Medical Sciences, Sirjan, Iran; 2 Community Based Psychiatric Care Research Center, Shiraz University of Medical Sciences, Shiraz, Iran; 3 Behavioral Sciences Research Center, Life Style Institute, Baqiyatallah University of Medical Sciences, Tehran, Iran; 4 Nursing Faculty, Baqiyatallah University of Medical Sciences, Tehran, Iran; 5 Community Based Psychiatric Care Research Center, School of Nursing and Midwifery, Shiraz University of Medical Sciences, Shiraz, Iran

**Keywords:** Elder abuse, Hospital, Mixed methods study, Questionnaire, Psychometrics properties

## Abstract

**Background:**

Older patients are more vulnerable and prone to abuse and neglect in hospitals and acute care settings. The present study aimed to develop and assess the psychometric properties of a questionnaire for screening abuse in hospitalized older adults.

**Methods:**

This study was conducted from October 2017 to September 2019 using the exploratory sequential mixed-methods research design. The participants were selected among those admitted to various wards of six teaching hospitals affiliated with Shiraz University of Medical Sciences, Shiraz, Iran. In the qualitative phase of the study, using the inductive content analysis method, the concept of abuse in hospitalized older adults was extracted through individual in-depth semi-structured interviews with 16 older patients and 11 family caregivers. Based on qualitative findings and a review of existing literature, an initial version of the questionnaire was developed. In the quantitative phase of the study, the psychometric properties (face, content, construct, and convergent validity; internal consistency and stability) of the questionnaire were examined.

**Results:**

Based on qualitative findings and literature review, a pool of 154 candidate items was defined. These items were reduced to 37 after initial refinement, qualitative and quantitative face and content validity, and item analysis. The outcome of principal component analysis further reduced the number of items to 27, which were grouped into 5 components, namely “Shortcomings in management and care facility”, “Neglect of professional commitments”, “Physical and psychological abuse”, “Protracted treatment process”, and “Invasion of privacy”. The explained variance of these 5 components was 50.09% of the overall variability of the questionnaire. The convergent validity of the questionnaire was acceptable (*P* < 0.00, *r* = − 0.44). Cronbach’s alpha coefficient and intraclass correlation coefficient for the entire questionnaire were 0.89 and 0.92, respectively; indicating high reliability and stability of the questionnaire.

**Conclusion:**

The hospitalized elder abuse questionnaire (HEAQ) has acceptable psychometric properties. It is recommended to use HEAQ to screen for suspected cases of abuse of hospitalized older adults.

**Supplementary Information:**

The online version contains supplementary material available at 10.1186/s12877-022-03400-0.

## Background

Elder abuse (EA) is a worldwide public health issue that negatively affects the human rights of many older adults. It is predicted that EA will rise in line with the rapid growth in the population of older people [1]. Many researchers, clinicians, and health policymakers have acknowledged EA as a major threat to the health and well-being of older adults [2]. This social phenomenon can occur within families as well as therapeutic relationships in hospitals and institutional settings such as nursing homes, residential care, and daycare facilities [3, 4]. However, one of the main concerns in taking care of older patients is the occurrence of EA in hospitals and institutional settings [4–6]. Although many studies have been conducted on EA in community settings, little attention has been paid to abuse in institutional settings where older patients are more vulnerable and prone to abuse [1]. Also, there are virtually no theory-based explanations of what causes institutional abuse and neglect of older adults [7]. Evidence shows that the reported prevalence of domestic EA is lower compared to institutional homes and care facilities [3]. The reported prevalence of institutional EA is heavily underestimated and amounts to only 20% of the true prevalence. Staff self-report data at various institutions (e.g., nursing homes, assisted living, residential care institutions, residential facilities, health facilities, and skilled nursing facilities) indicate that the overall prevalence of abuse of older residents is 64.2% [8]. There are few studies on the abuse of older patients in acute care settings. Work stress and burnout among staffs are the main reasons for increased EA in acute care settings. In addition, staff shortage does not only lead to inadequate staff screening and subsequent recruitment of underqualified staffs, but also results in poor supervision and management [9]. In Iran, like many developing countries, there is no accurate estimate of the prevalence of various types of EA since the monitoring and reporting of its occurrence is not the responsibility of one specifically designated organization [10].

Maintaining patients’ respect and dignity are essential components of the Patients’ Rights Charter [11]. However, older patients are at a higher risk of patients’ rights violation in hospitals due to the nature of their illness, clinical environment, and the behavior of medical teams [12]. Those with a physical disability, dementia, or cognitive impairment require a higher level of care, but at the same time are in a higher risk of abuse because of their aggressive behavior due to disability or impairment [9, 13]. Older patient vulnerability to abuse and rough handling is exacerbated by factors such as the type, severity, and duration of illness or injury, the associated diagnostic or therapeutic interventions, and higher dependency levels. Rough treatment by the clinical care team is characterized by threats and bullying, impatient and unreliable staff, ignorance, and treatment of patients as objects rather than people. Ageist attitudes of hospital staff further exacerbate the vulnerability of such patients and consequently expose them to aggression during care [14]. A previous study reported that 30% of older patients have experienced age discrimination in hospitals [15].

Previous studies have shown that older patients and persons with dementia or cognitive declines are more prone to abuse in healthcare settings [16, 17]. Another study reported that the abuse of hospitalized older adults could have immediate psycho-emotional consequences (feeling of insecurity and aggression) or serious and persistent consequences (learned helplessness) [18].

Cases of abuse in health care, despite the possibility of serious suffering to patients, have not been fully studied [19]. It has been shown that abuse becomes a normal practice when remains unnoticed, ignored, tolerated, accepted, or reinterpreted [20]. Given the extent of EA and its devastating impact on the victims, families, society, and the healthcare system. It is therefore important that regular and effective EA screening is given the highest priority in health centers and that health professionals regularly perform it to detect EA and continue to improve the quality of care [21].

EA is a hidden phenomenon, i.e., hidden by victims, their families, and perpetrators alike. It involves a wide range of behaviors that are rarely reported to the authorities. Usually, the main reason for not reporting the abuse is because of the cognitive impairments of older patients, making it impossible to report the abuse [22]. Other barriers include fear of consequences by victim or perpetrator, social or financial dependence on the perpetrator, fear of losing or worsening the relationship with the perpetrator resulting in unpredictable consequences, fear of being blamed; shame and embarrassment, self-blame, low self-confidence and self-esteem, physical frailty; low social status, financial weakness, the stigma associated with seeking help, feelings of ambivalence, anxiety, sense of loss and helplessness; inability to prevent abuse, doubts about the capability and adequacy of social workers to help them, a lack of trust in health professionals and their availability; a lack of effective social support, isolation, fear of not being taken seriously, perceiving abuse as a minor issue not worthy of disclosure; and cultural, generational, or religious beliefs [23]. In terms of reporting EA by health professionals, the main barriers include ambiguities about the definition, identification, and reporting of a case of abuse [24]; absence of a reporting system, challenges associated with the diagnosis of abuse; insufficient knowledge and training to assess, handle, and report cases of abuse; neglect of older patients, tendency not to confirm cases of abuse or verify suspected cases; a lack of protocol to identify abuse, fear of accepting responsibility and the desire not to confront abuse or get involved in such matters [25, 26], time constraint and insufficient resources to support abuse victims, limited available services to verify abuse and implement diagnosis [26], victim’s request not to report abuse, ageism and negative attitude toward older adults, tendency of victims to isolate themselves from physicians or health care providers; subtle and nonspecific clinical presentation (e.g., poor hygiene or dehydration), and the fear of harming their relationship with the hospital or care center [25].One of the main challenges in detecting EA, particularly in institutional care settings, is the lack of a reliable screening instrument [27]. It is therefore essential to devise strategies to improve diagnostics and reduce abuse and neglect of older adults [28]. In addition, the lack of a reliable screening tool is one of the main challenges in detecting EA particularly in institutional care settings [27]. Proper investigation and detailed reporting of such events using a reliable and standardized screening tool are essential for effective screening of EA [29].

The prerequisite for any successful EA intervention is the availability of effective screening and diagnostic tools. However, the hidden nature of the abuse poses a serious challenge to such diagnoses [22]. This underscores the need for a reliable and validated EA screening instrument to help practitioners to identify and screen EA in different settings. Nevertheless, despite several studies, assessment of EA is an understudied subject and development of screening tools stagnates. This is partly due to the lack of clarity and consensus about the basic definition of abuse, insufficient and incomplete data on the prevalence and incidence of EA, inadequate empirical research on the development and psychometric assessment of EA tools, divergent conceptions of causation, and insufficient funding to develop such tools [30]. Although a variety of direct and indirect screening and diagnostic methods have been developed, there is still a need for further research to develop and refine EA tools based on scientifically valid methods and by involving multidisciplinary teams. Based on various tools and methods, certain progress has however been achieved in the area of screening and diagnosis of EA, in both community and institutional settings [22]. Among commonly used instruments to screen for domestic EA are the 12-item Vulnerability to Abuse Screening Scale (VASS), the 52-item Abuse of the Elderly in the European region (ABUEL), the 41- and 44-item Elder Abuse and Neglect Assessment Instrument (EAI), the Indicators of Abuse (IOA) that includes 46 items for older patients and 44 items for caregivers, the 22-item Geriatric Mistreatment Scale (GMS), the 5-item Brief Abuse Screen for the Elderly (BASE), the 15-item Hwalek-Sengstock Elder Abuse Screening Test (H-S/EAST), the 13-item Modified Caregiver Strain Index (MCSI), and the 8-item Caregiver Abuse Screen for the Elderly (CASE) [31]. However, these tools may not be suitable to investigate abuse in institutional settings. With these tools, some indicators of abuse are difficult to observe in institutional settings and some specific indicators of institutional abuse are either omitted or given low priority. For example, unlike indicators in community settings, indicators of physical abuse at institutional residential settings include slapping patients for refusal to eat, inappropriate use of physical restraints (straitjackets, posey restraint, a drawsheet tied to patient’s chest, geriatric chair restraints, bedside railings), or chemical restraints (use of sedative or psychotherapeutic drugs to prevent certain movements or behaviors) by personnel as a convenient alternative to monitor and/or treat the patients. Furthermore, indicators of physical neglect at institutions include failure or delay in responding to call bells/lights, negligence in providing effective medical care, failure to administer drugs, excessive use of sedatives, failing to change bed linen, and failure to loosen physical restraints, etc. Indicators of psychological abuse include threats to discharge the patient, relocation to another room for no reason, withholding services (care, medications, food), and creating a sense of fear of institutional authorities, personnel, and even other patients. Indicators of psychological neglect include staff creating a feeling of rejection, being left alone for hours or threatening to do so, reduced cognitive stimulation, unplanned activities that may agitate the patients, poor nurse-patient communication, and inappropriate physical environment (lack of stimuli, light, color, etc.). Indicators of material abuse include allegations of theft of personal effects (clothes, valuables), limited provision of facilities (lockable drawer, wardrobe, safety box), and misuse of financial resources. Violation of personal rights is another indicator of abuse in institutions that include depriving a patient of free choice, privacy, and decision-making. In addition, the staff may avoid consulting a patient about the admission process, proposed care plan, treatment decisions and other important topics (thus taking away a patient’s control over both major and routine issues), and breach patient confidentiality [32].

Available literature on EA has primarily focused on domestic violence and little research has been done on EA in institutional settings [33]. Therefore, there is a lack of understanding of the determinants and characteristics of institutional abuse [34, 35]. Due to the lack of a comprehensive screening tool, there are only limited studies on the identification of EA and its prevalence at long-term care institutions. As a direct result, the extent of institutional abuse has not been fully understood [36]. It is therefore essential to develop valid and reliable tools to address this issue and gain a better understanding of institutional EA. Several tools have been developed to assess EA; however, these are mainly used for interviewing the staff of long-term care facilities. Moreover, they are designed to obtain an overall perspective of medical staff rather than the concerns of older patients subjected to abuse. For example, Pillemer and Moore used the Conflict Tactics Scale (CTS) to assess physical and psychological abuse of older patients in nursing homes. Hsieh et al. developed the Caregiver Psychological Elder Abuse Behavior (CPEAB) scale with a specific focus on identifying EA by the staff in nursing homes [37]. Cooper et al. also developed the 16-item Care Home Conflict Scale (CHCS) to screen for EA by the staff in nursing homes [38]. Other similar tools include the 25-item single-factor Elder Abuse Questionnaire (EAQ) by Kutowitz and Bowling, the 32-item Elders’ Psychological Abuse Scale (EPAS) by Wang et al., and the Minimum Data Set Assessment Instrument for Home Care (MDS-HC) by Morris et al. The EPAS is developed to determine psychological EA abuse in long-term care facilities and home settings. The MDS-HC is a multidimensional instrument that assesses the occurrence of EA. It is designed for use by clinical experts (to be completed at home or in a nursing home) through interviewing older patients and it only includes 5 items related to EA [31]. Overall, most studies on EA in long-term care facilities have used different instruments without reporting any psychometric assessments or have only reported partial information [37, 39]. They also do not evaluate the effectiveness of the tool used in identifying the risk of abuse in older patients residing in long-term care facilities and, therefore, many cases of abuse cannot be detected [40]. More importantly, these tools are not designed to assess EA by the staff in acute care centers such as hospitals.

In addition, screening tools should generally be fit-for-purpose and target the environment for which they are used [30]. The context in which abuse occurs is a determinant factor [41]. Given the complex nature of abuse and its nonspecific clinical presentation, EA cannot be treated as a typical disorder [25]. Therefore, it is unrealistic to expect a single EA instrument to fulfill all the requirements for the different settings, e.g., community and institutions [25, 30]. Hence, proposing an instrument for all types of abuse and settings is not practicable. The need for a dedicated instrument for a specific group and setting remains [38]. It is a fact that the context of EA differs in different settings and the nature of abuse in the community and family differs from those in acute and long-term care centers. Various studies have addressed abuse in the community as well as long- and short-term care centers. However, acute care centers such as hospitals have not been studied. Given the above, the prime focus of the present study is to assess the abuse of older patients in hospitals.

Considering the limited scope of the available screening tools, various studies have indicated the need for further research to develop a valid and reliable tool to detect and assess EA [27, 29, 32, 42–44]. Moreover, a previous study emphasized the need for the inclusion of cultural sensitivities as an important and necessary step in developing such tools [45].

To the best of our knowledge, there are no tools available to screen for abuse by health care personnel against hospitalized older adults. There is no question that caring for older adults in today’s acute care centers can be a challenging and complex process. Their unique needs are not always obvious and can be easily overlooked, which in turn leads to loss of patient dignity [9], vulnerability to abuse, and harsh treatment [14]. In addition, staff who are not trained to deal with aggressive and demanding behavior of older patients may unknowingly mistreat them by providing improper care, patronizing them, or causing severe physical and emotional harm. To preserve the dignity of older patients, it is therefore important to devise strategies for the identification, prevention, and mandatory reporting of EA cases [9].

Also according to a previous study, risk factors such as abuse, exploitation, and dissatisfaction negatively affect patients’ trust in their physicians, which may discourage older adults from seeking help and treatment [46]. This is also the case when a patient has a negative experience from previous hospitalizations [9]. Some studies have indicated that abuse in health care centers may even result in a total loss of trust in the entire healthcare system [47–49], to the extent that patients may avoid or at best delay seeking treatment [48]. In another study, Berglund et al. reported that patients’ suffering is exacerbated when they feel distrusted, mistreated, or their own views on illness and health are ignored during treatment; causing patients to feel mistrusted and rejected by the staff [50]. Considering all the above-mentioned timely detection of abuse against older patients is the first step in restoring their confidence in the healthcare system and regaining health. It is therefore essential to develop a tool that accurately assesses EA in hospital settings.

## Methods

### Study aim

The present study aimed to develop and assess the psychometric properties of a questionnaire for screening abuse in hospitalized older adults.

### Study design

This study was conducted from October 2017 to September 2019 using the exploratory sequential mixed-methods approach. In the absence of a reliable measuring tool, this method is particularly useful in developing and validating a new tool [51]. A norm-referenced approach, as proposed by Waltz et al., was used to define a conceptual model to outline the dimensions of the measurement process, describe the objectives of the tool in detail, develop a blueprint, and construct the tool including the categorization of items, scoring system, and assessment of the psychometric properties of the designed tool [52].

### Study population

The participants were selected among those admitted to various wards of six teaching hospitals affiliated with Shiraz University of Medical Sciences, Shiraz, Iran.

### Eligibility criteria

In the qualitative phase of the study (first phase), after obtaining permission from the authorities of each department, the eligibility of older patients was assessed based on their medical records and age. Initially, patients with acute clinical or emotional conditions (recently undergone surgery, severe pain, physical or psychological limitations, etc.) and those with restricted visitations were identified and excluded. Then, patients deemed fit to be interviewed were considered eligible. Using the purposive sampling method and based on the clinical judgment of the first author, some of these patients were selected as potential candidates for the study. Subsequently, only patients with rich experience in EA during current or previous hospitalization as key informants were included. Similarly, family caregivers were recruited based on the judgment of the first author, provided that the inclusion criteria were met and that they had first-hand experience with hospitalized older adults. Overall, the selection process of older patients and family caregivers was challenging due to the lack of specific protocols to identify EA in acute care settings.

In the quantitative phase, only hospitalized older adults were enrolled in the study based on the same inclusion criteria as in the qualitative phase. Conditional to their willingness to complete the questionnaire, these eligible patients participated in different stages of the psychometric assessment of the instrument.

Inclusion criteria for older patients for both qualitative and quantitative phases were aged ≥60 years, current hospitalization for at least 3 days, physical/mental/psychological ability to participate in an interview and respond to the questionnaire, willingness to participate, and having a good command of the Persian language. The exclusion criteria for these patients for both qualitative and quantitative phases were unwillingness to participate, incomplete questionnaire in the quantitative phase, acute conditions (e.g., pre- or post-surgical issues, delirium), and the likelihood of additional stress, emotional and psychological problems as a result of their participation in the interviews or while completing the questionnaire. In terms of family caregivers, they only participated in the qualitative phase of the study. The inclusion criteria for these participants were age ≥ 18 years, having accompanied the patient for a minimum of 3 days during the current hospitalization, capacity to provide in-depth information, physical/mental/psychological ability to participate in an interview, and willingness to participate. The exclusion criterion was withdrawal from the study.

### Qualitative methods

In the qualitative phase of the study, using the inductive content analysis method, the categories and sub-categories associated with the concept of abuse against hospitalized older adults were extracted through 29 individual in-depth semi-structured interviews (16 older patients and 11 family caregivers (two participants were interviewed twice). However, some of the older patients were unable to undergo the interview process since they were unconscious or had cognitive impairments. Given the importance of the perspective of these vulnerable patients on abuse and neglect for the objectives of our study, we instead interviewed their family caregivers. As customary in Iran, family caregivers are permitted to be physically present at the patient’s bedside and, consequently, as key informants, are fully aware of the potential abuse and neglect of their patient and even other patients in the hospital. They became the voice of those patients who could not participate due to their acute conditions. We have no doubt that these groups of family caregivers contributed positively to the identification and clarification of unknown aspects of EA. Moreover, since we aimed to design an instrument that would cover all aspects of abuse and neglect, from the perspective of both older patients with acute or extremely weak conditions as well as those who were slightly healthier, we effectively approached two groups of family caregivers as key informants. To avoid developing an instrument that could potentially miss important aspects of EA, we decided not to evaluate these groups separately. Details of the selection process of the participants in the qualitative phase and the interview guide for both older patients and family caregivers are described in our previously published research [5].

The items of the questionnaire were essentially based on the codes and sub-categories derived from the qualitative data analysis. The objective for each category was then determined based on a detailed description of each category and its association with the concept of EA. Subsequently, a blueprint was developed based on which the sub-categories associated with each category were defined and evaluated to improve the wording of the items of the questionnaire related to that category. Finally, a set of related and appropriate items corresponding to each category of the concept of EA was defined. In this stage, a pool of candidate items was defined in accordance with operational definitions and based on the extracted categories and sub-categories. Finally, the items of the questionnaire were determined on the basis of qualitative findings (inductive) and a review of existing literature as well as comparable tools (deductive).

### Quantitative methods

Based on the data obtained from the qualitative phase, the questionnaire was designed and its psychometric properties were assessed in the quantitative phase. To collect the required data for the face/construct validity and reliability of the instrument, we visited various wards in hospitals affiliated with Shiraz University of Medical Sciences. After describing the objectives of the study to hospital authorities and obtaining their permission, all eligible older patients were approached and written informed consent was obtained. Voluntary participation, the right to withdraw from the study, and confidentiality of any disclosed information were emphasized to the patients. Given that many older adults in Iran are illiterate, the questionnaire was completed using both the self-report and interview method to obtain comprehensive data representative of hospitalized Iranian older adults. All literate patients completed the questionnaire themselves while first author was present to answer any questions. In the case of illiterate patients, the first author read each question out loud completely and clearly and their scores were recorded in their presence. Such a difference in the manner of completing the questionnaire could however be considered a limitation of our study.

Face validity of the questionnaire was measured using both the qualitative and quantitative approaches. Qualitative face validity was conducted through face-to-face structured interviews with 15 hospitalized older adults meeting the same inclusion criteria as in the qualitative phase. Each item of the questionnaire was assessed for relevance, ease of response, and ambiguity [53] and adapted accordingly. Then, qualitative face validity was established by determining the importance of each item using the item impact method. To do so, another group of 15 hospitalized older adults was requested to score the importance of each item based on a 5-point Likert scale, according to which items with impact scores < 1.5 were removed from the questionnaire [54].

Content validity of the questionnaire was also examined using both the qualitative and quantitative approaches. Qualitative content validity was conducted by requesting 15 experts specialized in the field of nursing, geriatrics, and tool design to evaluate the questionnaire in terms of grammar, wording, essentiality, importance, item allocation, scaling, simplicity, and clarity [52, 55]. The content validity ratio (CVR), content validity index (CVI), and scale-level content validity index (S-CVI) were examined in the quantitative phase. The essentiality of each item was scored by the experts based on a 3-point Likert scale (1 = essential, 2 = useful but not essential, or 3 = not essential). Based on the Lawshe table (minimum value of CVR for 15 experts agreeing on which items are essential), items with CVR > 0.49 were considered essential and important (*P* > 0.05) [56, 57]. The relevancy of each item was measured by assessing CVI using a 4-point Likert scale, based on which items with CVI > 0.78 [57]. were kept in the questionnaire. After calculating the CVI for each item, the probability of chance agreement (P_C_) was first computed using the binomial random variable formula:

$${\mathrm{P}}_{\mathrm{C}}=\left[\mathrm{N}!/\mathrm{A}!\left(\mathrm{N}-\mathrm{A}\right)!\right]\times {0.5}^{\mathrm{N}}$$

where N = the number of experts, A = the number of agreeing on good relevance.

Then, the modified kappa (κ) coefficient was computed using the proportion of agreements on relevance (i.e., CVI) or the probability of P_C_:

$$\text{K}=\left(\mathrm I-\mathrm{CVI}-\mathrm{Pc}\right)/\left(1-\mathrm{Pc}\right)$$

κ value between 0.4 and 0.59 is considered fair, between 0.6 and 0.74 is considered good, and above 0.74 is considered excellent. The S-CVI was calculated based on the average scores of the CVI of all items of the questionnaire. Initially, the CVI for each item (I-CVI) was computed based on which the average of all I-CVI values was determined (S-CVI/Ave) for the entire questionnaire. A S-CVI/Ave value of 0.9 is considered an excellent criterion and a value of 0.8 as the lower content validity limit for the acceptance of the entire tool [58, 59].

Before evaluating the construct validity, an initial reliability test (item analysis) with a sample size of 50 hospitalized older adults was conducted to pilot the questionnaire. Cronbach’s alpha coefficient, inter-item correlation, and item-total correlation were calculated to determine those items having the least correlation with the concept or items that affect reliability [60]. Typically, inter-item correlations in the range of 0.3 to 0.7 are preferred [61].

Principal component analysis (PCA) and varimax rotation methods were used to evaluate the construct validity. These methods are the most common orthogonal rotation methods to reduce uncorrelated data. As a rule of thumb, a minimum of 300 samples is required to perform PCA based on which 301 hospitalized older adults were enrolled to assess the construct validity of the questionnaire. Initially, Bartlett’s test of sphericity was performed to determine the correlation between variables. Also, Kaiser-Meyer-Olkin (KMO) sampling index test was performed to measure sampling adequacy. A scree plot showing the eigenvalues was used to determine the number of components to retain in the PCA [62].

In the absence of any standardized or comparable scale to score EA of hospitalized older adults, convergent validity was used to correlate our designed questionnaire with those in the literature with confirmed convergent validity [63, 64]. For this purpose, the Persian version of the 12-item Short Form Health Survey (SF-12) questionnaire with confirmed validity and reliability was used [65]. The SF-12 and our designed questionnaire were simultaneously filled in by 100 hospitalized older adults and the correlation coefficient between the two was calculated.

Reliability assessment of the designed questionnaire was performed by determining the internal consistency using Cronbach’s alpha (a sample size of 50 older patients) and relative and absolute stability using the test-retest method (a sample size of 32 older patients) quantified with the intraclass correlation coefficient (ICC) and standard error of measurement (SEM). Responsiveness, interpretability, user-friendliness, and the scoring system of the questionnaire were also determined. The responsiveness of the questionnaire was determined using the COSMIN classification (Consensus-based Standards for the selection of health status Measurement Instruments), SEM according to Polit and Yang, and the minimum detectable change (MDC or SDC) benchmark [61, 66]. Measurement error was determined using the standard deviation of the difference between test and retest scores as well as the intraclass correlation coefficient (ICC). In addition to MDC, the percentage of MDC was also computed to determine the true relative changes between repeated measurements over time and to identify the relative amount of measurement random error. An MDC% below 30 is considered acceptable, and an MDC% below 10 is considered excellent [67]. The interpretability of the questionnaire was determined using the floor and ceiling effect and the distribution of total scores in the samples (a sample size of 301 older patients). The user-friendliness of the questionnaire was determined using two criteria, namely the response rate and the percentage of respondents not answering each item.

Quantitative data were analyzed using SPSS software (version 23.0).

## Results

In the qualitative phase of the study, 29 individual in-depth semi-structured interviews were conducted to define the concept of EA in hospitalized older adults. The qualitative data were classified based on the inductive content analysis method proposed by Elo and Kyngäs (2008). Accordingly, the concept of the abuse of hospitalized older adults and its dimensions were established as follows: “The abuse of hospitalized older adults is a multi-factorial and multi-dimensional phenomenon. In addition to individual and professional factors, issues related to the inadequate physical environment and organizational structure of hospitals have drastically contributed to the occurrence of EA. The abuse includes physical and emotional abuse at the personal level, the neglect of both the patients and professional duties, unethical behavior as well as the presence of an unsafe environment and confusing conditions for older patients. On top of these issues, shortcomings in the organizational structure, by management and in policy (e.g., the cumbersome process from admission to discharge, limited financial resources, and financial abuse of the patients) have a negative impact on the treatment of older patients” [5]. The main categories of this concept were classified into micro-level, meso-level, exo-level, and macro-level issues. A detailed description of qualitative data analysis, categorization, and dimensions of the abuse of hospitalized older adults has already been presented in our article on the qualitative phase [5].

Initially, a draft version of the questionnaire contains a pool of 154 candidate items was formulated based on the findings from our qualitative phase [5]. The initial pool of items was in a raw format without any modifications or psychometric assessments (e.g., face or content validity, factor analysis). The items were reviewed independently by each member of the research team followed by a group review. Then, in a joint meeting, each item of the pooled data was examined and duplicated items were removed. To gain confidence in the results and reduce potential bias by examiners, the raw data and selected items were presented to two external experts for independent evaluation. The feedback from the experts was then discussed and reviewed in a joint meeting of the research team. In three iterations, the initial 154 items were reduced to 106 items, then 91 items, and finally to 73 items. In this process, we mainly focused on reducing items that were either repetitive or could be merged with each other. At this stage, no psychometric assessment was performed. Potential disagreements between team members were resolved by referring to the research supervisor and the items were further reviewed independently by each team member followed by a joint meeting. This process was repeated until a full consensus was reached.

Eventually, 73 items were classified into 4 main categories and 11 generic categories, namely micro-level issues (28 items), meso-level issues (21 items), exo-level issues (12 items), and macro-level issues (12 items). These were then used to assess the psychometric properties of the designed questionnaire. Since more codes were identified in the qualitative phase of the study, the micro-level and meso-level issues included a higher number of items. During the assessment process of the psychometric properties of the 73-item questionnaire, some items were removed at different stages of the face validity and content validity of the questionnaire.

Qualitative face validity of the 73-item questionnaire resulted in the modification of 10 items (in terms of writing style, wording, and readability) and 3 items were merged with other questions. The resulting 70 items were used in the quantitative face validity stage after which 3 items with impact scores < 1.5 were removed, reducing the total number of items to 67 questions.

In the qualitative content validity stage, based on the feedback from the expert panel, 3 overlapping items were merged with similar items and 44 words were modified. The CVR of the remaining 64 items was then computed. Based on the Lawshe table (minimum CVR for 15 experts), 19 items with CVR ≤ 0.49 were removed. The CVI of the remaining 45 items was > 0.78 with an excellent kappa coefficient (> 0.74), thus no items were removed. Based on the average scores of the CVI of all items of the questionnaire, the calculated S-CVI was 0.95 (acceptable). The inter-item correlation coefficient was in the range of 0.3 to 0.7, and thus no items were removed or merged. However, 8 items with item-total correlation < 0.3 were removed, reducing the total number of questions to 37 items. Before evaluating construct validity, the initial reliability of the questionnaire was 0.88.

Construct validation of the 37-item questionnaire was conducted using a sample size of 301 older patients. The mean age of the participants was 68.3 ± 7.81 and the mean hospital stay in the emergency department or other appropriate wards was 2.81 ± 4.40 and 8.47 ± 9.89, respectively. Of all older patients, 55.1% were male, 80.1% married, 45.8% illiterate, and 54.2% literate. The adequacy of sampling was confirmed based on the significance level of Bartlett’s test (*P* < 0.001) and KMO index > 0.8 [55]. A factor loading of 0.3 was considered as the minimum acceptable degree of correlation between each item and the extracted factors. The results showed that the explained variance of the 5 components was 50.09% of the overall variability of the questionnaire (Table 1).

**Table 1:** The variance explained by each component before and after rotation using the principal component analysis method

Components	Eigenvalues			The sum of squared factor loadings before rotation			The sum of squared factor loadings after rotation		
	Total	% of variance	Cumulative %	Total	% of variance	Cumulative %	Total	% of variance	Cumulative %
1	5.67	21.00	21.00	5.67	21.00	21.00	3.82	14.15	14.15
2	2.83	10.49	31.49	2.83	10.49	31.49	2.68	9.93	24.08
3	1.96	7.26	38.76	1.96	7.26	38.76	2.63	9.75	33.84
4	1.57	5.83	44.59	1.57	5.83	44.59	2.48	9.19	43.03
5	1.48	5.49	50.09	1.48	5.49	50.09	1.90	7.05	50.09

Accordingly, 27 items associated with these components were selected and the remaining 10 items were removed. In line with the content of the 27 items of the questionnaire, the components were labeled as “Shortcomings in management and care facility” (8 items), “Neglect of professional commitments” (9 items), “Physical and psychological abuse” (4 items), “Protracted treatment process” (3 items), and “Invasion of privacy” (3 items) (Table 2). The process of item reduction from 154 to 27 is presented in Table 3.

**Table 2:** The five components extracted using varimax rotation and the corresponding loading factor for each item

Components	Items		Principal components analysis				
			Extracted factors				
			1	2	3	4	5
**Shortcomings in management and care facility**	1	Have you been provided with basic necessities (e.g., gown, clean bed sheets, blanket, chair, slippers, etc.)?	0.52				
	2	Have you had access to medical supplies when needed (pressure mattress, ambulance, walker, wheelchair, adjustable electric bed, etc.)?	0.40				
	3	Does the size of your hospital room correspond to the number of its patients?	0.75				
	4	How do you rate the quality of the indoor air of your hospital room in terms of freshness, scent, and ventilation?	0.77				
	5	Is the air temperature in your hospital room satisfactory?	0.64				
	6	Did the hospital respect quiet hours at night (lighting and noise)?	0.75				
	7	Are your hospital room and bathroom maintained clean and sanitized?	0.72				
	8	Are your hospital room, bathroom, corridors age-friendly (adequate grab rails, non-slip flooring, accessibility, etc.)?	0.69				
**Neglect of professional commitments**	9	Have your visitation rights ever been restricted without any medical reasons?		0.58			
	10	Did you ever had to plead with the medical staff for care or treatment?		0.58			
	11	Has the medical team ever refused to properly address your pain?		0.57			
	12	Have you ever felt being neglected or ignored by the medical team?		0.71			
	13	Have you ever felt being excessively charged by the hospital (unnecessary hospital stay, repetitive tests, chaperone fees, provision of excessive amounts of medical supplies, etc.)?		0.52			
	14	Have you experienced arbitrary cancelation of your surgery or test even after hours of fasting?		0.31			
	15	Have you ever felt anxious because no medical information was provided?		0.46			
	16	Have you ever been denied assistance with personal needs in the ward (eating meals, bathing, dressing, short walk, etc.)?		0.50			
	17	Was the medical team ever indifferent to your discomfort and suffering?		0.46			
**Physical and psychological abuse**	18	Have you ever been addressed by the hospital staff in an angry or aggressive manner?			0.76		
	19	Have you ever been insulted or disrespected by the hospital staff?			0.80		
	20	Have you or your family caregivers ever experienced acts of violence by hospital staff?			0.72		
	21	Have you ever been blamed by the hospital staff for any accidents?			0.55		
**Protracted treatment process**	22	Typically, how long did you have to wait for treatment?				0.56	
	23	Have you ever had to wait for admission because there was no bed available on the ward?				0.85	
	24	Has the discharge process from the emergency unit or transfer to a ward been efficient and timely?				0.81	
**Invasion of privacy**	25	Have the hospital staff ensured there was no unnecessary exposure of parts of your body during clinical examination?					0.78
	26	Did the medical staff ask for your permission before physical inspection of the most private areas of your body?					0.79
	27	Did the hospital staff respected your religious beliefs?					0.56

**Table 3:** The process of item reduction following initial reviews by the research team and psychometric assessment

Stage	Items (n)	Items reviewed/merged/removed (n)	Items kept (n)
Initial pool of items, review by the research team: First stage	154	48	106
Review by the research team: Second stage	106	15	91
Review by the research team: Third stage	91	18	73
Qualitative face validity	73	Face-to-face interview with 15 older patients. 3 items merged and 10 items reviewed and edited.	70
Quantitative face validity (impact score)	70	3 items with impact score < 1.5 removed	67
Qualitative content validity	67	3 items merged and 44 items reviewed and edited.	64
Quantitative content validity, content validity ratio (CVR)	64	19 items with CVR < 0.49 removed	45
Quantitative content validity, content validity index (CVI)	45	No items removed: CVI > 0.79, kappa coefficient > 0.74	45
Item analysis	45	8 items with item-total correlation coefficient < 0.3 removed	37
Construct validity (principal component analysis)	37	10 items removed due to factor loading < 0.3	27
Final questionnaire	27		

Evaluation of convergent validity showed an inverse relationship and moderate correlation between the scores obtained from our questionnaire and the SF-12 questionnaire (*P* < 0.001, *r* = − 0.44). In other words, the higher the EA score the lower the health-related quality of life of the hospitalized older adults. In addition to SF-12, convergent validity between our questionnaire and a general question put to older patients (“How would you rate the overall care you received from the hospital? Please rate from 0 to 100”) was assessed. The results showed an inverse relationship and strong correlation between the two (*P* < 0.001, *r* = − 0.77). It indicated that the higher the EA score, the significantly lower the satisfaction with the conduct of hospital staff and quality of care provided. Based on the above, the convergent validity of the designed questionnaire was confirmed.

The results of internal consistency with a Cronbach’s alpha coefficient of 0.89 showed high reliability of the designed questionnaire. The coefficient for each of the 5 components of the questionnaire was between 0.71 and 0.87. The stability of the questionnaire was assessed using the test-retest method. A total of 32 older patients filled in the questionnaire twice at an interval of 7 to 10 days. In the first round, the patients completed the questionnaire on day 1 and the questionnaire was completed for the second time after a minimum of 7–10 days of hospitalization. The minimum requirement for the test-retest interval was 7 days. According to a previous study, the time interval between the two tests should be long enough (e.g., 1 or 2 weeks) to prevent the participants to remember their previous responses and to eliminate potential changes in the observed behavior that could affect repeatability [38]. Generally, due to usual illnesses among older people, their length of hospital stay is longer than other patients [68]. Since some of our patients were hospitalized for more than 30 days, we used the hospital medical records to ensure that only those with an expected minimum of 7 days of hospitalization could participate. In addition, for the retest, we evaluated these patients again according to our inclusion criteria. Those with acute conditions, delirium due to the lengthy hospital stay, or unwilling to continue with the study were excluded. Anticipating a high dropout rate, we choose to enroll not less than 100 patients from different wards for the first round of test-retest assessment. Indeed, 68 patients did not participate in the second round, and thus the test-retest assessment was performed with the remaining 32 older patients.

The scores from each test were used to calculate the ICC for each category and the entire questionnaire. ICC coefficient ≥ 0.80 indicates satisfactory stability (23, 26). The results showed that the ICC coefficient for each category and the entire questionnaire was between 0.85 and 0.95, confirming the stability of the designed questionnaire (Table 4).

**Table 4:** Intraclass correlation coefficient (ICC) between test-retest scores of the entire questionnaire

Component		Mean ± SD	ICC	95% confidence interval		***P*** value
				Infimum	Supremum	
1	Shortcomings in management and care facility	20.59 ± 6.38	0.93	0.85	0.97	< 0.001
2	Neglect of professional commitments	20.76 ± 5.76	0.85	0.63	0.93	< 0.001
3	Physical and psychological abuse	5.57 ± 2.19	0.95	0.87	0.97	< 0.001
4	Protracted treatment process	9.43 ± 2.87	0.95	0.90	0.98	< 0.001
5	Invasion of privacy	6.15 ± 2.02	0.90	0.79	0.95	< 0.001
Total questionnaire		60.53 ± 14.45	0.92	0.70	0.96	< 0.001

The results of the measurement error of the entire questionnaire confirmed a degree of absolute stability (Table 5).

**Table 5:** Measurement error and comparison MDC and percentage of MDC for the components and the entire questionnaire

Component		Score range	SEM	MDC	%MDC	Status ^a^
1	Shortcomings in management and care facility	40–8	1.68	4.65	22.58	Acceptable
2	Neglect of professional commitments	45–9	2.23	6.18	29.77	Acceptable
3	Physical and psychological abuse	20–4	0.48	1.33	23.87	Acceptable
4	Protracted treatment process	15–3	0.64	1.77	18.76	Acceptable
5	Invasion of privacy	15–3	0.63	1.74	28.39	Acceptable
Total questionnaire		135–27	4.08	11.30	18.66	Acceptable

^a^ Minimum detectable change percentage: < 30% = Acceptable, < 10% = Excellent

Also, the floor and ceiling effect showed that the minimum and maximum scores for the entire questionnaire were zero. The distribution of total scores in the samples indicated a significant direct relationship between EA of older adults during a normal hospital stay and stay in an emergency department (*P* < 0.001). However, this relationship was not present for other variables (age, sex, hospital stay in other units, marital status, employment status, and living conditions) (*P* > 0.05). Nonetheless, the mean EA score was different for the level of education, i.e., older patients with a higher level of education perceived abuse more than those with lower education. The least significant difference (LSD) post-hoc test showed that older patients with a university degree perceived abuse more significantly compared to other groups (*P* < 0.001). Also, compared to other groups, those older patients who perceived a higher degree of abuse did not wish to be referred to the same hospital under any circumstances in the future (*P* < 0.001). The average time for older patients to fill in the questionnaire by themselves or with the help of a research team member (through an interview) was 15 minutes (range: 10 to 20 minutes) and 22.5 minutes (range: 20 to 25 minutes), respectively. The percentage of respondents not answering all items of the questionnaire was < 5%.

The questionnaire is scored based on a 5-point scale. Positively phrased items (3, 4, 7, 9–20) are awarded 5 points for the “always/ very much” response and 1 point for “never/ not at all”. Whereas items (1, 2, 5, 6, 8, 21–27) are scored in reverse and awarded 5 points for “never/ very slow/ absolutely inappropriate” response and 1 point for “always/ very timely/ absolutely appropriate”. The total score ranges from 27 to 135 and is categorized as mild abuse (from 27 to 63), moderate abuse (from 64 to 99), and severe abuse (from 100 to135) (Additional file 1).

## Discussion

In the present study, a questionnaire was designed to assess EA in hospitalized older adults. The design included parameters such as Iranian cultural sensitivities, the perception, experience, and expectation of the older patients, and variations in the organization and resources of health centers (e.g., hospitals) across Shiraz (Iran). Evaluation of psychometric properties confirmed the validity (content, face, construct) and reliability of the designed questionnaire.

Among tool developers, it is generally agreed that the content of any tool must be derived directly from relevant respondents [69], fit for purpose, and applicable to the intended setting [30]. Since the context in which abuse occurs is a determinant factor [41], it is likely that EA screening tools do not cover all settings-related aspects of abuse within a specific community or institution [30]. For instance, investigation of EA in institutional settings [acute care centers or long-term care facilities] requires addressing the underlying issues within care systems and interpersonal dynamics. An EA screening tool is subject to severe limitations without a comprehensive study of older adults’ lived experiences, psychosocial aspects, and the effect of social context on abuse [70]. Screening tools targeting elderly abuse at institutions (e.g., acute care centers due to their specific mission) are therefore required to include interpersonal dynamics in interactions as well as context and construct differences associated with EA in such challenging settings. Therefore, in the qualitative phase of the study, we directly approached older patients and their family caregivers to obtain their perceptions and experiences of EA in order to develop appropriate questions.

Overall, the evidence shows conceptual ambiguities in the screening of EA. Currently available EA screening tools (abuse in long-term care settings and domestic abuse) are either poorly defined or lack validity and reliability assessment. Even those that have fulfilled certain aspects of validity and reliability requirements are frequently adapted by researchers to fit the purpose of their study. Hence finding a comprehensive EA screening tool has been a challenging task [41]. Some researchers have resorted to tools developed using quantitative approach based on theories derived from various forms of domestic abuse (e.g., child abuse or violence against women). Clearly, these do not comprehensively address the issue of screening domestic EA. Moreover, the validity and reliability of only a few such tools have been confirmed [45]. On the other hand, currently available tools to screen for domestic EA tend to focus on specific and more evident indicators such as physical abuse or exploitation. With these tools, screening for potential negligence or detecting sub-categories of EA is a real challenge [27, 30].

Key factors that affect the validity and reliability of a screening tool are the number of items in a component, the extent to which these items are representative of the construct, and the coherence between the items. Most studies, particularly in the field of medical science, have relied on a single item per type of abuse which results in limited and potentially unreliable screening. In contrast, the use of multiple items allows identification of the more subtle aspects of abuse as well as assessment of the severity and frequency of its occurrence. Since EA is a latent and complex construct, a reliable screening tool should include multiple items, each guided by research and theory [41]. Some EA screening tools have only been tested using a small sample size which undermines their generalizability and sheds doubts on the construct validity of results. Additional concerns are too much focus on only one aspect of EA (i.e., domestic violence) and identification of its legal and clinical parameters. It is therefore recommended to further develop and refine screening tools in this area, particularly in the case of institutional abuse against older adults [27].

The main challenge faced by researchers in the field of EA is to find a standardized and user-friendly screening tool with confirmed validity and reliability [71]. There are several tools that examine EA in the family and community, namely Vulnerability to Abuse Screening Scale (VASS, Schofield & Mishra, 2003), Elder Assessment Instrument (EAI, Fulmer, 1984), Brief Abuse Screen for the Elderly (BASE, Reis et al., 1993), Indicators of Abuse (IOA, Reis & Nahmiash, 1998), Hwalek-Sengstock Elder Abuse Screening Test (H-S/EAST, 1986), Caregiver Abuse Screen for the Elderly (CASE, Reis & Nahmiash, 1995), and Domestic Elder Abuse Questionnaire (Heravi-Karimooi et al., 2010) [31, 43, 72–76]. To the best of our knowledge, only a few tools have been developed that primarily focus on institutional EA. However, they only addressed EA in long-term care settings (e.g., nursing homes, etc.) and did not cover acute care centers such as hospitals. Wang and colleagues developed and validated the Elders’ Psychological Abuse Scale (EPAS) to examine EA in long-term care facilities and home settings in Taiwan [77]. Kottwitz and Bowling developed the Elder Abuse Questionnaire (EAQ) and piloted it among the residents and personnel of a long-term care facility [78]. Ballard and colleagues (2017) developed a 9-item questionnaire called Elder Abuse Suspicion Index for Use in Long-Term Care (EASI-ltc) [36] and Hsieh et al., (2009) developed and examined the Caregiver Psychological Elder Abuse Behavior Scale (CPEAB) [79]. However, these tools were merely designed to examine abuse from the perspective of healthcare personnel or family caregivers rather than from the perspective of the elderly themselves. Moreover, the tools were not comprehensive as they only addressed one aspect of EA. Little has been published on the psychometric properties of these tools and thus there is still a lack of appropriate diagnostic tools dedicated to screening EA in various care facilities [36]. Except for the EPAS, other tools were designed and developed based on a review of the available literature, whereas the questionnaire in our study was developed using the inductive-deductive method. Moreover, these tools were designed to examine abuse in long-term care facilities (e.g., nursing homes) which is not applicable to acute care settings such as hospitals. Referring to the COSMIN guideline, assessment of psychometric properties of tools should include validity (content, criterion, construct), reliability (internal consistency, test-retest, inter-rater agreement, measurement error), responsiveness (sensitivity and ability to detect change), and interpretability (the degree of assigning qualitative meaning of the least significant changes to quantitative scores) [66, 80]. However, most of the designed tools, including those addressing domestic and long-term institutional abuse, lack certain key psychometric properties or the measured properties are incomplete [33, 37, 39].

As a final note, one should recognize that the topics of EA assessment and developing an appropriate tool are still in their infancy stage. Due to the lack of clarity on the definition of EA and due to limited empirical research into the development and testing of screening tools, this field of study requires refinement. It is therefore important that new tools are developed based on both a theoretical framework and accurate cognitive testing methods. Since screening is the first phase of any investigation, EA tools should be developed to detect various forms of EA and assessed accordingly. As mentioned, there is no comprehensive and definitive tool to detect EA. Currently available tools, both abuse in long-term care settings and domestic abuse, merely detect a possible case of abuse for referral to the respective authorities for detailed investigation. Nonetheless, these tools will enable the detection of a potential EA in a systematic, standardized, and multidisciplinary manner. Therefore, it is essential that these tools are comprehensively assessed for the possibility of incorporating modifications and their validity and reliability have to be confirmed [27].

One of the main limitations of our study was the reluctance of some older patients to fully disclose all details of their personal experiences of abuse; possibly because of the fear of repercussions by the staff they depend on for their treatment and care. This in turn resulted in partial completion of the questionnaire by a number of older patients and thus a more limited description of EA. Also, the generalizability of our findings is hindered since the first phase of the study was qualitative and the participants were recruited only from Shiraz (Iran). Data triangulation was used to resolve the generalizability issue, including persons (semi-structured interviews with older patients and their family caregivers), time (conducting interviews during different hospital shifts), and space (different units in various hospitals). Moreover, in addition to the qualitative findings on which we based the formulation of the questionnaire items, we included the results of a large number of high-quality scientific publications on EA and abuse in health care settings in our research. On a positive note, the findings of our study and the designed questionnaire provide a comprehensive package of information on EA in hospitalized older adults which can be used as a basis to promote theoretical development and support future research. It can also facilitate the timely detection of EA across health systems. Further studies are recommended to confirm the validity and reliability of the designed questionnaire in both public and private hospitals as well as using a larger elderly population with different cultural backgrounds. Another limitation of this study was the lack of specific protocols to identify EA in acute care settings. Also, the difference in completing the questionnaire (self-report by literate patients and through an interview by the first author for illiterate patients) was a limitation in our study. However, we reduced the effect of this limitation by enrolling a similar number of literate (54.2%) and illiterate (45.8%) patients.

## Conclusion

A 27-item questionnaire is developed to address elder abuse in hospitals. It encompasses the perception, experience, and expectation of older patients as well as the cultural, social, and religious context of Iranian society. Evaluation of psychometric properties confirmed the validity (face, content, construct) and reliability of the designed questionnaire. The questionnaire is short, user-friendly, and can be used for self-reporting by older patients or completed through an interview with patients. As the main feature, the questionnaire allows the detection of various types of EA by institutional caregivers or medical personnel that may occur during the hospitalization of older adults.

## Supplementary Information

**Additional file 1.**

## Data Availability

The datasets used and analyzed during the current study are available from the corresponding author on reasonable request.

## Abbreviations

HEAQ: Hospitalized elder abuse questionnaire
EA: Elder abuse
CVR: Content validity ratio
CVI: Content validity index
S-CVI: Scale-level content validity index
S-CVI/Ave: Scale-level content validity index/average
P_C_: Probability of chance agreement
PCA: Principal component analysis
KMO: Kaiser-Meyer-Olkin
SF-12: 12-item short-form health survey
ICC: Intraclass correlation coefficient
SEM: Standard error of measurement
COSMIN: Consensus-based standards for the selection of health status measurement instruments
MDC: Minimum detectable change
SDC: Smallest detectable change
SPSS: Statistical package for the social sciences
