# Supplementary material for: Development and psychometric properties of the hospitalized elder abuse questionnaire (HEAQ): a mixed methods study
Source: BMC Geriatr. 2022 Aug 30;22:715. doi: 10.1186/s12877-022-03400-0 (PMC9426014; doi:10.1186/s12877-022-03400-0)
Supplement: Supplementary file 1 — Additional file 1. [file 12877_2022_3400_MOESM1_ESM.docx]

**Appendix I**

| During your current hospitalization: | | Always | Very Often | Sometimes | Rarely | Never |
| --- | --- | --- | --- | --- | --- | --- |
| 1 | Have you been provided with basic necessities (e.g., gown, clean bed sheets, blanket, chair, slippers, etc.)? |  |  |  |  |  |
| 2 | Have you had access to medical supplies when needed (pressure mattress, ambulance, walker, wheelchair, adjustable electric bed, etc.)? |  |  |  |  |  |
| 3 | Have your visitation rights ever been restricted without any medical reasons? |  |  |  |  |  |
| 4 | Did you ever had to plead with the medical staff for care or treatment? |  |  |  |  |  |
| 5 | Have the hospital staff ensured there was no unnecessary exposure of parts of your body during clinical examination? |  |  |  |  |  |
| 6 | Did the medical staff ask for your permission before physical inspection of the most private areas of your body? |  |  |  |  |  |
| 7 | Has the medical team ever refused to properly address your pain? |  |  |  |  |  |
| 8 | Did the hospital staff respected your religious beliefs? |  |  |  |  |  |
| 9 | Have you ever felt being neglected or ignored by the medical team? |  |  |  |  |  |
| 10 | Have you ever felt being excessively charged by the hospital (unnecessary hospital stay, repetitive tests, chaperone fees, provision of excessive amounts of medical supplies, etc.)? |  |  |  |  |  |
| During your current hospitalization: | | Very much | Much | Somewhat | Little | Not at all |
| 11 | Typically, how long did you have to wait for treatment? |  |  |  |  |  |
| 12 | Have you ever had to wait for admission because there was no bed available on the ward? |  |  |  |  |  |
| 13 | Have you experienced arbitrary cancelation of your surgery or test even after hours of fasting? |  |  |  |  |  |
| 14 | Have you ever felt anxious because no medical information was provided? |  |  |  |  |  |
| 15 | Have you ever been denied assistance with personal needs in the ward (eating meals, bathing, dressing, short walk, etc.)? |  |  |  |  |  |
| 16 | Have you ever been addressed by the hospital staff in an angry or aggressive manner? |  |  |  |  |  |
| 17 | Have you ever been insulted or disrespected by the hospital staff? |  |  |  |  |  |
| 18 | Have you or your family caregivers ever experienced acts of violence by hospital staff? |  |  |  |  |  |
| 19 | Have you ever been blamed by the hospital staff for any accidents? |  |  |  |  |  |
| 20 | Was the medical team ever indifferent to your discomfort and suffering? |  |  |  |  |  |
| During your current hospitalization: | | Very timely | Timely | Somewhat Timely | Slow | Very slow |
| 21 | Has the discharge process from the emergency unit or transfer to a ward been efficient and timely? |  |  |  |  |  |
| During your current hospitalization: | | Absolutely appropriate | Appropriate | Neutral | Inappropriate | Absolutely inappropriate |
| 22 | Does the size of your hospital room correspond to the number of its patients? |  |  |  |  |  |
| 23 | How do you rate the quality of the indoor air of your hospital room in terms of freshness, scent, and ventilation? |  |  |  |  |  |
| 24 | Is the air temperature in your hospital room satisfactory? |  |  |  |  |  |
| 25 | Did the hospital respect quiet hours at night (lighting and noise)? |  |  |  |  |  |
| 26 | Are your hospital room and bathroom maintained clean and sanitized? |  |  |  |  |  |
| 27 | Are your hospital room, bathroom, corridors age-friendly (adequate grab rails, non-slip flooring, accessibility, etc.)? |  |  |  |  |  |
